# Urine Albumin-Creatinine Ratio (ACR) of Patients With Chronic Obstructive Pulmonary Disease (COPD): A Cross-Sectional Study

**DOI:** 10.7759/cureus.86974

**Published:** 2025-06-29

**Authors:** Souradip Laha, Amrut Mohapatra, Rajlaxmi Sarangi, Jyoti Prakash Sahoo, Saswat Subhankar

**Affiliations:** 1 Pulmonary Medicine, Kalinga Institute of Medical Sciences, Bhubaneswar, IND; 2 Biochemistry, Kalinga Institute of Medical Sciences, Bhubaneswar, IND; 3 Pharmacology, Kalinga Institute of Medical Sciences, Bhubaneswar, IND

**Keywords:** cat score, correlation analysis, end stage renal disease (esrd), fev1/fvc ratio, forced expiratory volume (fev1), forced vital capacity (fvc), gold stage, micro albuminuria, renal impairment, urine albumin creatinine ratio

## Abstract

Background and objectives

Chronic obstructive pulmonary disease (COPD) and chronic kidney disease (CKD) may coexist. Their co-occurrence and impacts on renal and pulmonary functions have not been explored in detail. We planned this study to evaluate urine albumin-creatinine ratio (ACR) levels among patients with COPD. We also assessed the correlation among age, urine ACR, forced expiratory volume in 1 second (FEV_1_), Global Initiative for Chronic Obstructive Lung Disease (GOLD) stage, and COPD Assessment Test (CAT) score of the participants.

Methods

This cross-sectional study was conducted at Kalinga Institute of Medical Sciences (KIMS), Bhubaneswar, India, from March 2023 to April 2025. The following parameters were noted from the case sheets after admission: age, serum creatinine, urine albumin, urine creatinine, urine ACR, FEV_1_, forced vital capacity (FVC), FEV_1_/FVC, GOLD stage, and CAT score. We correlated all these values. We used R software (version 4.4.3; R Foundation for Statistical Computing, Vienna, Austria) for data analysis.

Results

Three hundred fifty-seven patients were recruited for the study. The median age of the participants was 61.0 years (54.0-69.0 years). 233 (65.3%) participants were males. The median serum creatinine was 1.97 mg/dL (0.81-2.48 mg/dL). The study population's median urine ACR was 24.59 mg/g (10.83-50.13 mg/g). The median FEV_1_ was 46.79% (39.78-60.72%) of the predicted value. The median FEV_1_/FVC was 0.57 (0.53-0.60). According to the GOLD staging, most individuals fell into stages 2 (134, 37.5%) and 3 (156, 43.7%). The median CAT score was 18.0 (14.0-21.0). There were positive associations between GOLD stage and CAT score (r = 0.767, p < 0.001), age and GOLD stage (r = 0.673, p < 0.001), and age and CAT score (r = 0.622, p < 0.001). There were negative associations between GOLD stage and FEV_1_ (r = -0.945, p < 0.001), age and FEV_1_/FVC (r = -0.851, p < 0.001), FEV_1_ and CAT score (r = -0.755, p < 0.001), and age and FEV_1_ (r = -0.736, p < 0.001). The significant correlations with urine ACR were found with GOLD stage (r = 0.409, p < 0.001), CAT score (r = 0.310, p < 0.001), FEV_1_ (r = -0.357, p < 0.001), and FEV_1_/FVC (r = -0.229, p < 0.001).

Conclusion

Urine ACR and CAT scores were higher among the elderly population. With aging, FEV_1_ readings decreased. Our study showed weak correlations between the lung parameters and urine ACR.

## Introduction

Chronic obstructive pulmonary disease (COPD) is one of the most common non-communicable diseases in India [1,2]. COPD is associated with low socioeconomic status, smoking, infections, malnutrition, aging, occupational exposures, indoor and outdoor air pollution, and genetics [3-5]. These parameters can trigger vascular anomalies, loss of alveolar capillary endothelial cells, death of alveolar cells, and widening of the alveolar gap. These events contribute to the development of COPD [4-6].

Numerous comorbidities coexist with COPD, especially among elderly individuals [7]. These comorbidities affect the morbidity, mortality, and financial burden. Therefore, it is essential to manage the comorbid conditions [7,8]. Among elderly individuals, chronic kidney disease (CKD) is quite common. The co-occurrence of COPD and CKD has not been explored thoroughly [7,9]. The primary method for screening for albuminuria and CKD is urine ACR [10].

The pulmonary function test (PFT) is routinely done in COPD patients to gauge their pulmonary function [11]. COPD is diagnosed if the FEV_1_/FVC of any person is below 0.7. The classification of COPD relies on the predicted percentage of FEV_1_ [12,13]. The severity of COPD is frequently assessed with COPD Assessment Test (CAT) scores and Global Initiative for Chronic Obstructive Lung Disease (GOLD) stages [13-16]. The GOLD stages 1-4 (i.e., mild, moderate, severe, and very severe) correspond to FEV_1_ values of ≥ 80%, 50-79%, 30-49%, and ≤ 30% of the predicted values, respectively [12,13]. CAT scores of 0-10, 11-20, 21-30, and 31-40 indicate low, medium, high, and very high impacts of COPD, respectively [15,16].

Albuminuria is a known risk factor for renal dysfunction, end-stage renal disease (ESRD), and cardiovascular morbidity and mortality [17]. Patients with normoalbuminuria, microalbuminuria, and macroalbuminuria have urine albumin-creatinine ratio (ACR) <30 mg/g, 30-300 mg/g, and >300 mg/g, respectively [17,18]. Individuals with micro- or macroalbuminuria are prone to developing nephropathy [10,17]. Higher urine ACR values indicate significant renal impairment [18]. Our study aimed to evaluate the COPD patients' urine ACR, FEV_1_, and CAT scores. We also assessed the correlation among various pulmonary (i.e., FEV_1_, FEV_1_/FVC, severity per the GOLD stage, CAT score) and renal (i.e., serum creatinine and urine ACR) parameters.

## Materials and methods

This cross-sectional study was conducted from March 2023 to April 2025. The Institutional Ethics Committee of Kalinga Institute of Medical Sciences (KIMS), Bhubaneswar, India, granted us ethical approval (KIIT/KIMS/IEC/1197/2023) before commencing the study. All participants gave their written informed consent before their enrollment.

Study participants

Adult patients diagnosed with COPD, with FEV_1_/FVC < 0.7, were included. Patients with diabetes, hypertension, and any respiratory illness other than COPD were excluded.

Procedure

The sociodemographic (age, gender, body mass index (BMI), biomass fuel exposure, and history of smoking) and clinical parameters (urine ACR, FEV_1_, FEV_1_/FVC, CAT score, GOLD stage, serum creatinine, and urine ACR) were recorded from the case sheets. All these values were noted following the initial assessment in the hospital. The Kuppuswamy classification was used to classify the socioeconomic class [19]. The FEV_1_ values for the GOLD stages 1-4 (i.e., mild, moderate, severe, and very severe) are ≥ 80%, 50-79%, 30-49%, and < 30% of the predicted values, respectively [12,13]. Low, moderate, high, and very high impacts of COPD are indicated by CAT scores of 0-10, 11-20, 21-30, and 31-40 [15,16]. Urine ACR values of less than 30 mg/g, 30-300 mg/g, and greater than 300 mg/g indicate normoalbuminuria, microalbuminuria, and macroalbuminuria, respectively [17,18].

Statistical analysis

For this cross-sectional study, we used convenience sampling. We used the Shapiro-Wilk test to determine whether the data distribution was normal and identified it as non-parametric. Frequencies and percentages were used to summarize the qualitative data. The median and the interquartile range (IQR) were used to illustrate the quantitative data. We used the Kruskal-Wallis and Pearson's chi-square tests to assess quantitative and qualitative data. Pearson's correlation was used to evaluate the relationship between the different factors. The associations were shown with the correlation coefficient and 95% confidence interval (CI). For data calculation, R software (R Foundation for Statistical Computing, Vienna, Austria) version 4.4.3 was used [20]. A p-value of less than 0.05 was considered statistically significant.

## Results

The cross-sectional study was conducted from March 2023 to April 2025. We screened 786 patients with COPD. Three hundred two patients had diabetes. Ninety-six patients had hypertension. Thirty-one were on anti-asthmatic medications. The remaining 357 patients were analyzed. Their demographic and clinical parameters are shown in Table 1. The participants had a median age of 61.0 (54.0-69.0) years. Of the 357 participants analyzed, 233 (65.3%) were males. Biomass fuel exposure was present in 128 (35.9%) patients. The median value for serum creatinine was 1.97 mg/dL (0.81-2.48 mg/dL). The median urine ACR of the study population was 24.59 mg/g (10.83-50.13 mg/g). The median FEV_1_ was 46.79% (39.78-60.72%) of the predicted value. The median FEV_1_/FVC was 0.57 (0.53-0.60). Most participants belonged to stage 3 (156, 43.7%) and stage 2 (134, 37.5%) of the GOLD staging. The median CAT score was 18.0 (14.0-21.0).

**Table 1:** Demographic and clinical parameters of the study participants (n = 357) The categorical data was expressed as frequency and percentage. The continuous data was represented by median and interquartile range (IQR). The categorical and continuous data were analyzed with chi-square (ꭓ²) and the Kruskal-Wallis tests. BMI: body mass index, urine ACR: ratio of urine albumin to urine creatinine, FEV_1_: forced expiratory volume in 1 second, FVC: forced vital capacity, FEV_1_/FVC: ratio of FEV_1_ to FVC, GOLD stage: Global Initiative for Chronic Obstructive Lung Disease stage, CAT score: COPD Assessment Test score. The reference range for the urine albumin (immunoturbidometric method) is 0.0-2.0 mg/dL. The reference range for the urine creatinine (Jaffe method) is 28.0-217.0 mg/dL. The reference range for the urine ACR is 0.0-29.0 mg/g.

Parameter	Value
Age (years)	61.0 (54.0–69.0)
Age group	
≤ 50 years	56 (15.7%)
51-60 years	111 (31.1%)
61-70 years	117 (32.8%)
> 70 years	73 (20.4%)
Gender	
Male	233 (65.3%)
Female	124 (34.7%)
BMI (kg/m^2^)	28.38 (24.79–33.81)
Biomass fuel exposure	128 (35.9%)
Urine albumin (mg/dL)	1.35 (0.58–2.53)
Urine creatinine (mg/dL)	48.99 (33.75–78.13)
Urine ACR (mg/g)	24.59 (10.83–50.13)
Serum creatinine (mg/dL)	0.79 (0.71–0.91)
FEV_1_ (%)	46.79 (39.78–60.72)
FEV_1_/FVC	0.57 (0.53–0.60)
GOLD stage	
1 (mild)	22 (6.2%)
2 (moderate)	134 (37.5%)
3 (severe)	156 (43.7%)
4 (very severe)	45 (12.6%)
CAT score	18.0 (14.0-21.0)

**Figure 1:**
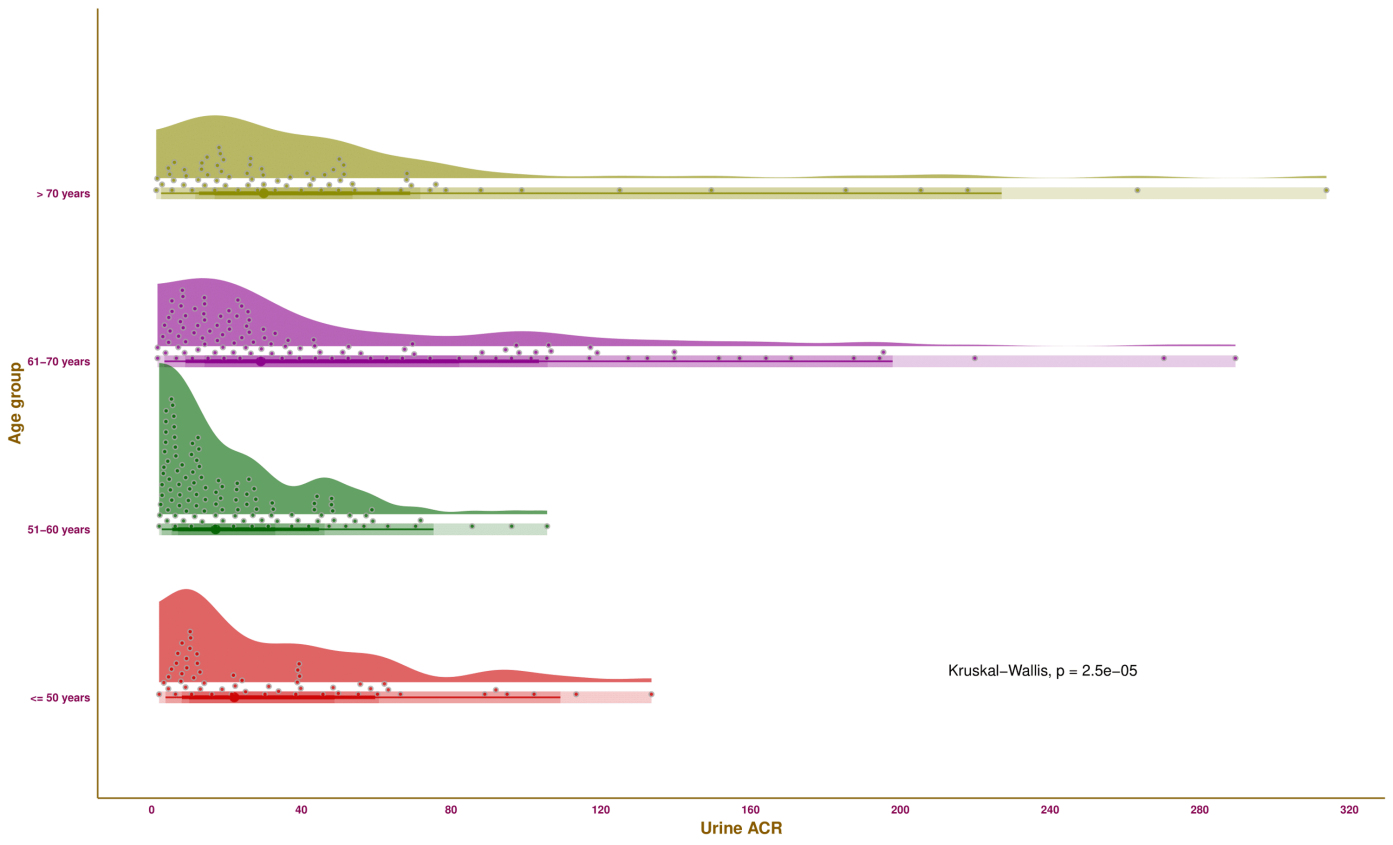
Urine ACR values of the study population The half-eye, interval, and beeswarm plots illustrate the urine ACR values (in mg/g) of the study participants of different age groups. The interval plot (the straight line) indicates the central 50%, 68%, 95%, and 100% (all) of the urine ACR values through different shades. Urine ACR: ratio of urine albumin to urine creatinine.

Figure 1 illustrates the urine ACR of the study participants of various age groups through half-eye, interval, and beeswarm plots. Each of the four groups was highlighted in distinct colours. The median values of urine ACR for the participants were as follows: 22.09 mg/g (10.03-48.83 mg/g) (those aged ≤ 50 years), 17.10 mg/g (7.09-33.02 mg/g) (those aged 51-60 years), 29.16 mg/g (14.15-42.18 mg/g) mg/g (those aged 61-70 years), and 29.96 mg/g (16.86-53.67 mg/g) (those aged > 70 years), respectively. The intergroup comparison yielded statistically significant differences (p < 0.001). The findings suggest that urine ACR values tend to increase with advancing age.

**Figure 2:**
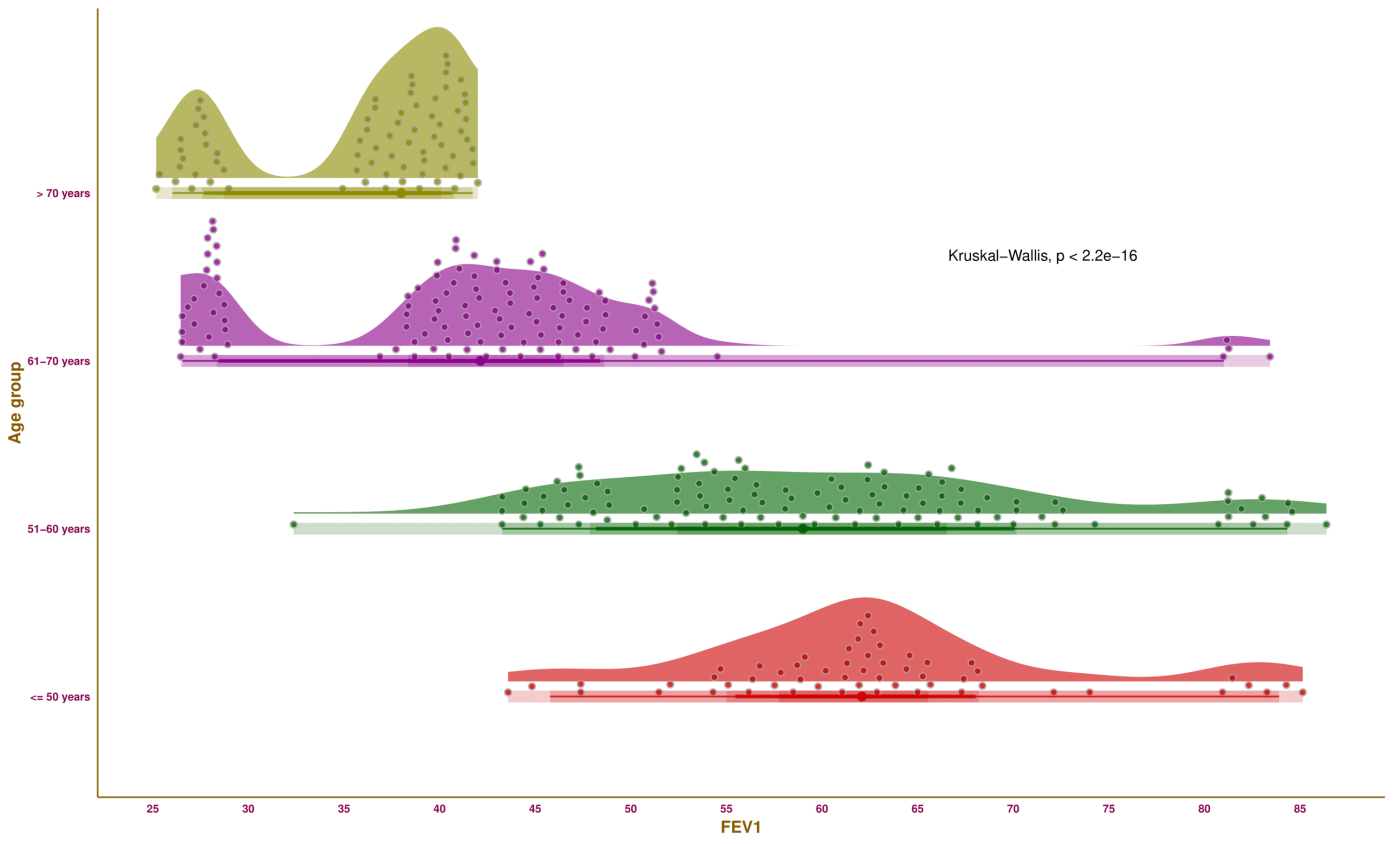
FEV1 values of the study population The half-eye, interval, and beeswarm plots illustrate the FEV_1_ values (in %) of the study participants of different age groups. The interval plot (the straight line) indicates the central 50%, 68%, 95%, and 100% (all) of the FEV_1_ values through different shades. FEV_1_: forced expiratory volume in one second.

Figure 2 illustrates the FEV_1_ of the study participants of various age groups through half-eye, interval, and beeswarm plots. Each of the four groups was highlighted in distinct colours. The median values of FEV_1_ for the participants were as follows: 62.09% (57.76-65.55%) (those aged ≤ 50 years), 59.00% (52.42-66.53%) (those aged 51-60 years), 42.14% (38.35-46.47%) (those aged 61-70 years), and 37.99% (28.72-40.09%) (those aged > 70 years), respectively. The intergroup comparison yielded statistically significant differences (p < 0.001). The findings suggest that pulmonary function tends to reduce with advancing age.

**Figure 3:**
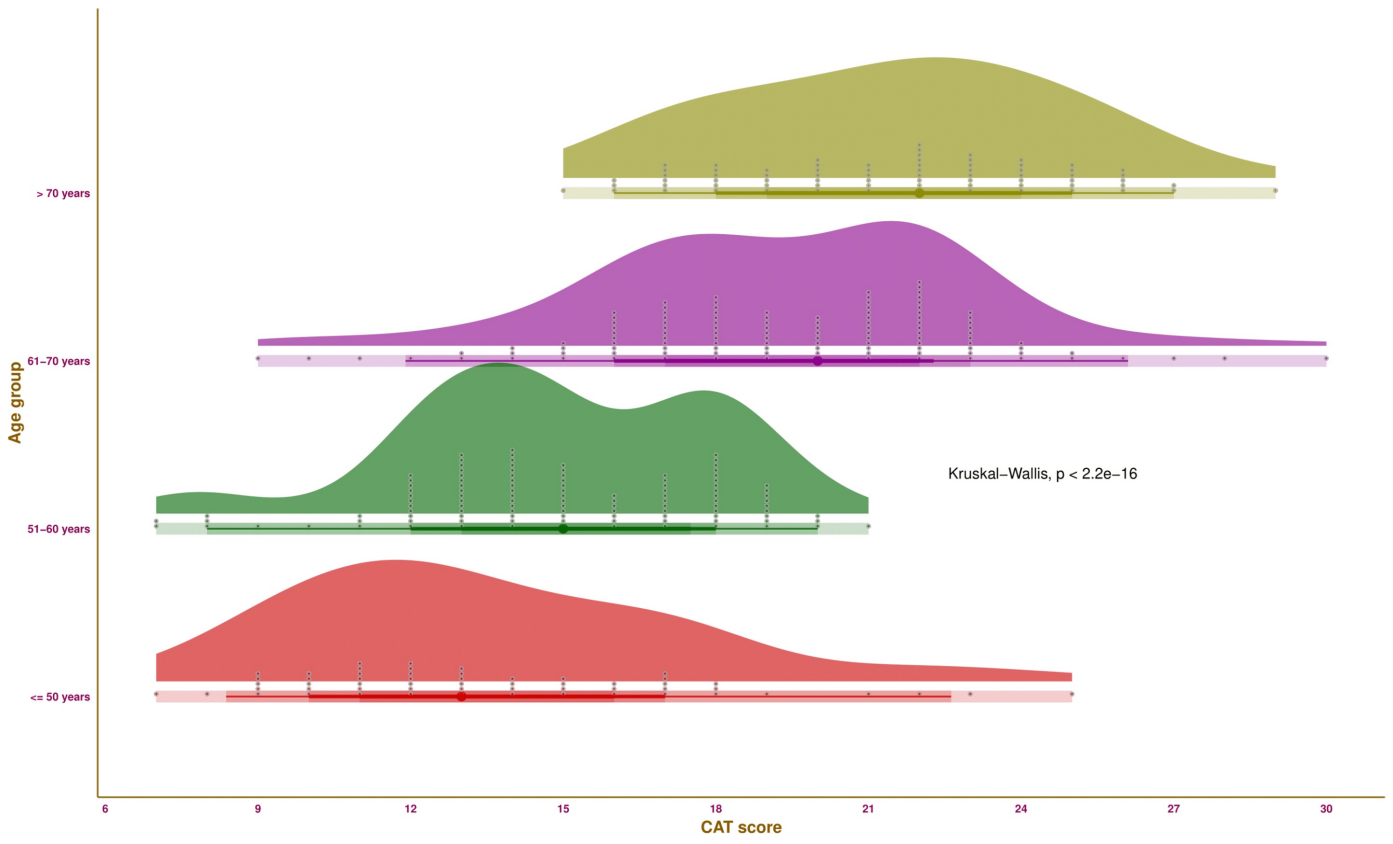
CAT scores of the study population The half-eye, interval, and beeswarm plots illustrate the CAT scores of the study participants of different age groups. The interval plot (the straight line) indicates the central 50%, 68%, 95%, and 100% (all) of the CAT scores through different shades. CAT: COPD Assessment Test.

Figure 3 illustrates the CAT score of the study participants of various age groups through half-eye, interval, and beeswarm plots. Each of the four groups was highlighted in distinct colours. The median values of CAT scores for the participants were as follows: 13.0 (11.0-16.0) (those aged ≤ 50 years), 15.0 (13.0-17.5) (those aged 51-60 years), 20.0 (17.0-22.0) (those aged 61-70 years), and 22.0 (19.0-24.0) (those aged > 70 years), respectively. The intergroup comparison yielded statistically significant differences (p < 0.001). The findings suggest that COPD tends to worsen with advancing age.

**Figure 4:**
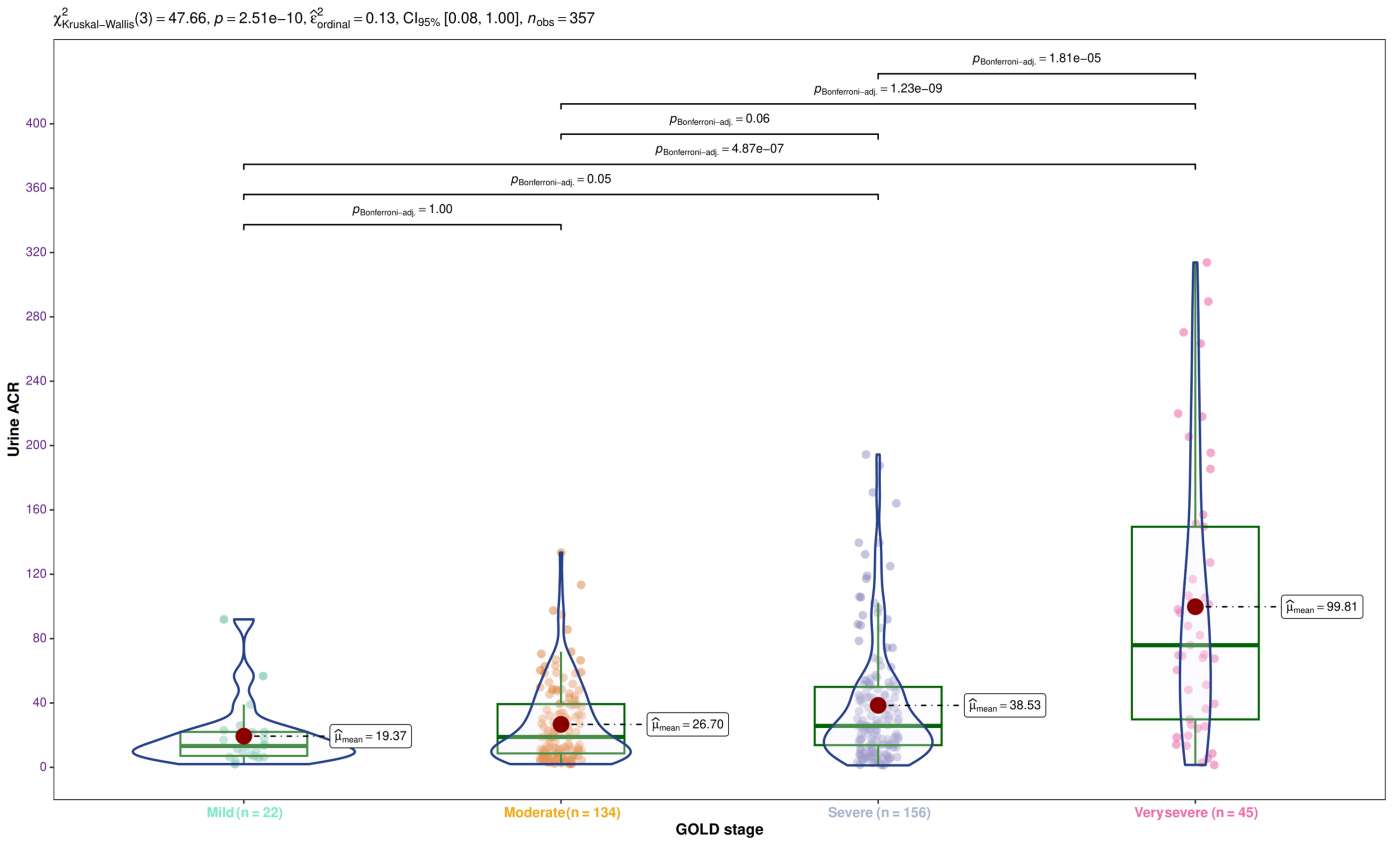
Distribution of urine ACR per GOLD stage The urine ACR values (in mg/g) are displayed via the violin, box-whisker, and jitter plots. The mean values are portrayed as the red dots. The Bonferroni test was leveraged following the Kruskal-Wallis test for the intergroup comparison. ACR: albumin-creatinine ratio; GOLD: Global Initiative for Chronic Obstructive Lung Disease

In Figure 4, we portrayed the urine ACR values of the participants with different severities of COPD. The violin, box-whisker, and jitter plots illustrate the dispersion of urine ACR values of the participants with mild, moderate, severe, and very severe COPD. The median urine ACR values for the corresponding groups were 13.25 mg/g (7.12-22.00 mg/g), 18.87 mg/g (8.66-39.33 mg/g), 25.75 mg/g (13.78-49.99 mg/g), and 75.92 mg/g (29.81-149.52), respectively (p < 0.001). The differences could be attributed to the duration of COPD and the age of the participants. The post hoc analysis with the Bonferroni test yielded statistically significant differences (p < 0.001) among participants with very severe COPD and milder forms.

**Figure 5:**
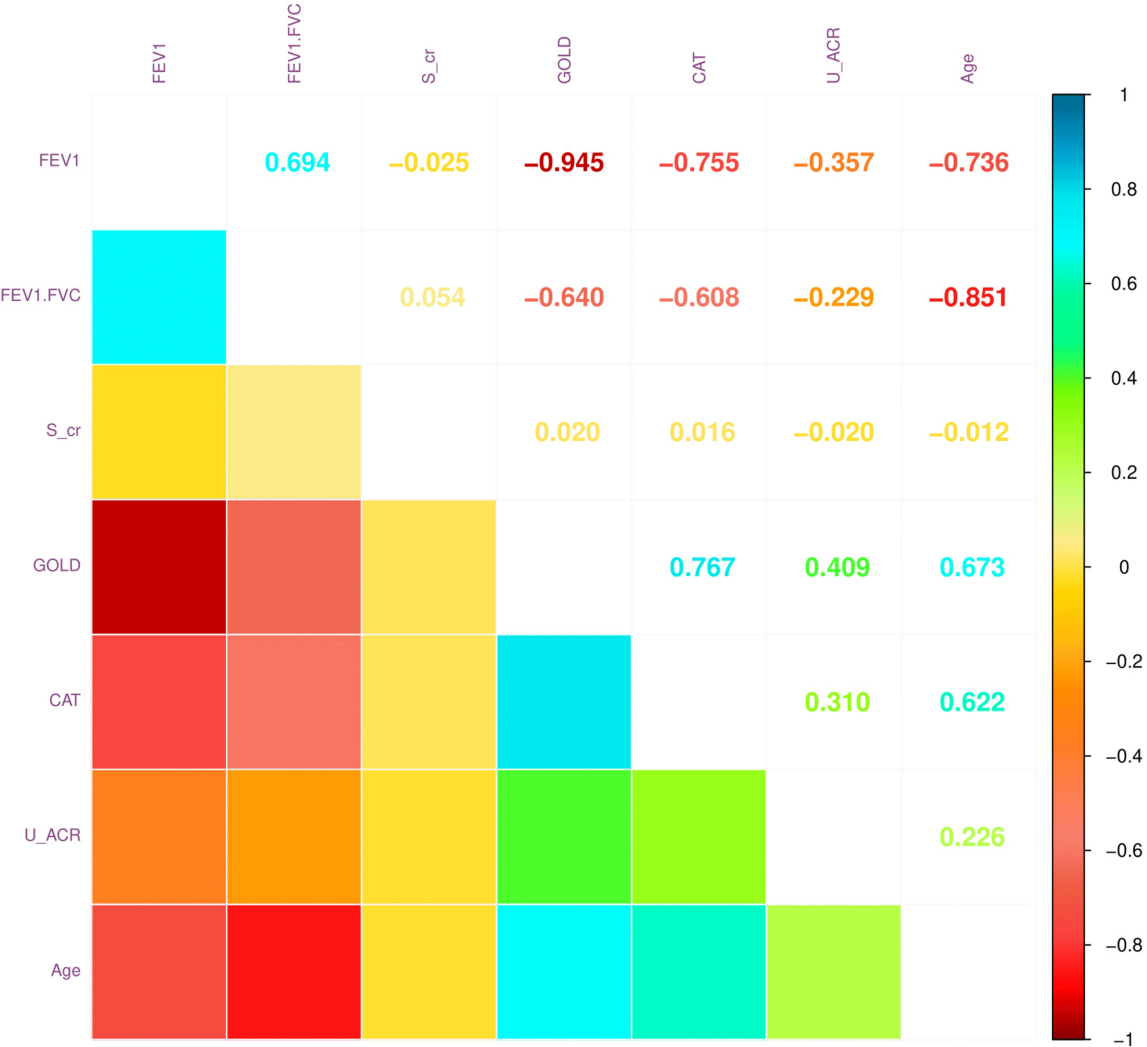
Correlation between pulmonary and renal parameters The correlation plot illustrates the degree of association among various pulmonary and renal parameters. The correlation coefficients range from -1 (strong negative correlation) to +1 (strong positive correlation). FEV_1_: forced expiratory volume in one second, FVC: forced vital capacity, FEV_1_/FVC: ratio of FEV_1_ to FVC, s_cr: serum creatinine, GOLD: global initiative for COPD, CAT: COPD assessment test, U_ACR: ratio of urine albumin to urine creatinine.

Figure 5 illustrates the correlation coefficients between various parameters. There were positive correlations between GOLD stage and CAT score (r = 0.767, 95% CI = 0.721 to 0.807, p < 0.001), FEV_1_ and FEV_1_/FVC (r = 0.694, 95% CI = 0.637 to 0.745, p < 0.001), age and GOLD stage (r = 0.673, 95% CI = 0.611 to 0.726, p < 0.001), and age and CAT score (r = 0.622, 95% CI = 0.554 to 0.681, p < 0.001). There were negative correlations between GOLD stage and FEV_1_ (r = -0.945, 95% CI = -0.955 to -0.933, p < 0.001), age and FEV_1_/FVC (r = -0.851, 95% CI = -0.877 to -0.820, p < 0.001), FEV_1_ and CAT score (r = -0.755, 95% CI = -0.796 to -0.706, p < 0.001), and age and FEV_1_ (r = -0.736, 95% CI = -0.780 to -0.684, p < 0.001). The significant associations with urine ACR were found with GOLD stage (r = 0.409, 95% CI = 0.371 to 0.448, p < 0.001), CAT score (r = 0.310, 95% CI = 0.286 to 0.335, p < 0.001), FEV_1_ (r = -0.357, 95% CI = -0.381 to -0.334, p < 0.001), and FEV_1_/FVC (r = -0.229, 95% CI = -0.254 to -0.204, p < 0.001). These findings suggested that the severity of COPD was not strongly associated with renal dysfunction.

## Discussion

In this study, we observed the renal and pulmonary parameters of the patients with COPD. The pulmonary parameters, like FEV_1_, FVC, and FEV_1_/FVC, were obtained through spirometry. The renal parameters, like serum creatinine, urine albumin, urine creatinine, and urine ACR, were tested in the laboratory after admission. We noted all these values from the case sheets of the 357 participants. There was a male preponderance (233, 65.3%) in the study population. The median age was 61 years. One-third of participants (128, 35.9%) had a history of biomass fuel exposure. The majority of patients had normoalbuminuria. Two hundred ninety participants (81.2%) had COPD of GOLD stages 2 and 3. The median CAT score of 18.0 suggested that the impact of COPD was moderate.

Nicotine contributes to lung tissue damage in COPD and triggers renal impairment. Moreover, it raises blood pressure by stimulating the sympathetic nervous system [7,21]. As a result, the prevention of oxidative damage by superoxide dismutase and catalase dwindles. Through reactive oxygen species (ROS), the substances in smoke may cause endothelial dysfunction and renal impairment [7,8]. Additionally, hypoxia may cause kidney damage. The high oxygen demand of the proximal convoluted tubule triggers its fibrosis due to hypoxia [22]. Hypercapnia in patients with COPD causes reduced renal blood flow, renal afferent arteriolar vasoconstriction, and sympathetic overactivation [7]. Age is another factor contributing to the gradually declining renal function in patients with COPD [8]. The senescent cells are affected by insults like smoke particles or inflammation. The advancing age also reduces the homeostatic processes and tissue healing mechanisms [7,8]. Moreover, many nephrotoxic drugs also deteriorate renal function in elderly patients [7].

FEV_1_/FVC provides a clearer illustration of respiratory ailments like COPD than the FEV_1_ value [12]. FEV_1_/FVC < 0.7 is required for the diagnosis of COPD. Then, the GOLD staging is done based on the FEV_1_ values [12,13]. The CAT score weighs the impact of COPD [15,23]. Urine ACR is the urine albumin concentration to urine creatinine concentration [18]. We assessed the correlation among these pulmonary parameters with age and urine ACR. The findings suggested an increasing trend of urine ACR. However, the correlation was not strong enough to illustrate any clinical difference. The FEV_1_ values had a decline pattern with age. The CAT scores had a rising trend with advancing age. The associations of age with FEV_1_ (r = -0.736, 95% CI = -0.780 to -0.684, p < 0.001) and CAT score (r = 0.622, 95% CI = 0.554 to 0.681, p < 0.001) were strong. We analyzed the urine ACR values per the participants' GOLD stages. The urine ACR values for those with very severe COPD (i.e., GOLD stage 4) were significantly higher as compared to those with milder forms of COPD. Studies by Gupta et al. [24] and Shayo et al. [25] suggested that urine ACR values increase with advancing stages of COPD.

Our study had a few shortcomings. First, our study's findings are not as broadly applicable because of the single study site. Second, the impact of various medications and comorbidities was not considered. Third, routine follow-ups could provide information about the long-term effects of renal impairment. Fourth, we did not include patients with diabetes or other respiratory conditions outside COPD.

## Conclusions

We evaluated the renal and pulmonary parameters of the patients with COPD. We also correlated urine ACR values with the severity of COPD (gauged with the GOLD stage), CAT scores, FEV_1_, and FEV_1_/FVC. Our study findings revealed that urine ACR values and CAT scores increased with the patient's age, and FEV_1_ values reduced with age. We found weak associations of urine ACR with the pulmonary parameters. However, we could not deduce the impact of urine ACR values on the progression of COPD. Hence, we recommend further prospective studies with a larger sample size and longer follow-up duration to gauge the association of renal and pulmonary parameters.
